# Perspectives of Pharmacy Students on Ethical Issues Related to Artificial Intelligence: A Comprehensive Survey Study

**DOI:** 10.21203/rs.3.rs-4302115/v1

**Published:** 2024-04-30

**Authors:** Hisham E. Hasan, Deema Jaber, Omar F. Khabour, Karem H. Alzoubi

**Affiliations:** Jordan University of Science and Technology; Zarqa University; Jordan University of Science and Technology; University of Sharjah

**Keywords:** Artificial intelligence, pharmacy education, ethics, technology literacy, MENA

## Abstract

**Background:**

The integration of artificial intelligence (AI) into pharmacy education and practice holds the potential to advance learning experiences and prepare future pharmacists for evolving healthcare practice. However, it also raises ethical considerations that need to be addressed carefully. This study aimed to explore pharmacy students’ attitudes regarding AI integration into pharmacy education and practice.

**Methods:**

A cross-sectional design was employed, utilizing a validated online questionnaire administered to 702 pharmacy students from diverse demographic backgrounds. The questionnaire gathered data on participants’ attitudes and concerns regarding AI integration, as well as demographic information and factors influencing their attitudes.

**Results:**

Most participants were female students (72.8%), from public universities (55.6%) and not working (64.2%). Participants expressed a generally negative attitude toward AI integration, citing concerns and barriers such as patient data privacy (62.0%), susceptibility to hacking (56.2%), potential job displacement (69.3%), cost limitations (66.8%), access (69.1%) and the absence of regulations (48.1% agree), training (70.4%), physicians’ reluctance (65.1%) and patient apprehension (70.8%). Factors including country of residence, academic year, cumulative GPA, work status, technology literacy, and AI understanding significantly influenced participants’ attitudes (*p* < 0.05).

**Conclusion:**

The study highlights the need for comprehensive AI education in pharmacy curricula including related ethical concerns. Addressing students’ concerns is crucial to ensuring ethical, equitable, and beneficial AI integration in pharmacy education and practice.

## Introduction

The landscape of higher education and pedagogy is undergoing a profound transformation with the growing prevalence of online learning, a shift accelerated by the COVID-19 pandemic [1]. As society becomes increasingly digitized, the adoption of various digital tools, including artificial intelligence (AI), has become a prominent trend in shaping the educational experience [2, 3]. The quest for quality education, aligned with the United Nations (UN) sustainable development goal for 2030, has seen the integration of digital innovations that co-create information, mentor, and assess [4]. One prominent model in this evolving landscape is hybrid education, also known as blended learning. Hybrid education integrates synchronous and asynchronous teaching modes for online and offline students, allowing student autonomy in planning and acquiring their education [4].

Among the emerged AI technologies in adaptive learning is ChatGPT [5]. This tool offers personalized, real-time support by understanding user intent and aiding with course-related queries. While such technologies enhance accessibility and engagement, challenges include contextual understanding, reliance on external data, and potential bias. Ethical concerns about plagiarism, overdependence on AI for critical thinking, and the impact on educational norms have also been raised by educators and researchers [6, 7].

AI is revolutionizing healthcare by aiding in precision and personalized medicine, predicting treatment responses, optimizing drug doses, and minimizing adverse events [8]. The rapid integration of AI in healthcare education is driven by students’ positive perceptions and demand for AI incorporation in their training [9]. Global professional pharmacy organizations’ recommendations, like those by the International Pharmaceutical Federation, emphasize the importance of incorporating digital health and AI instruction into pharmacy curricula [10]. This growth of AI raises questions about challenges and strengths, particularly in education. Privacy breaches and misinformation are vulnerabilities that must be addressed, while the potential strength lies in leveraging technology to counter such issues. Future pharmacists must integrate AI ethically into patient care, collaborate with AI-driven tools, and contribute to healthcare system enhancements.

Although the ethical considerations surrounding the evolving role of teachers and the potential disruption to traditional teaching methods have been discussed, concerns about the impact of AI on pharmacy students’ enthusiasm for future pharmacy practice and education exist. In response to the evolving role of AI in pharmacy education, this study aims to delve into the perspectives of pharmacy students regarding the ethical issues surrounding AI integration. By comprehensively examining the challenges and strengths students perceive, this research seeks to inform educational approaches that can effectively address concerns and facilitate the ethical integration of AI into the ever-evolving landscape of pharmacy practice and education.

## Material and Methods

### Study Design

This study employed a cross-sectional design to investigate the perspectives of pharmacy students on ethical issues related to integrating AI into their education. A validated online questionnaire, using “Google Forms”, served as the primary tool for data collection, which took place from August 2022 to January 2023.

### Questionnaire Development

The questionnaire (Additional file 1) was developed collaboratively with pharmacy practice and research ethics experts and grounded in a comprehensive literature review [11–13]. Comprising two parts, the first section gathered sociodemographic information from the students, while the second section focused on collecting responses regarding pharmacy students’ perspectives on AI integration. The five-point Likert scale, ranging from "strongly disagree = 1" to "strongly agree = 5", was used to gauge participant opinions. The questionnaire language was simple Arabic. All questions on the survey platform are designed as mandatory responses to prevent the possibility of missing data.

### Sample Size and Selection

The minimum required sample size was determined using Raosoft, with a 95% confidence level and a 5% margin of error. It was found to be 377 students. The target population included pharmacy students from diverse educational institutions in the MENA (Middle East and North Africa) region. Participants were recruited online, employing a convenience sampling technique via social media platforms (WhatsApp, Facebook, and LinkedIn groups).

### Ethical Considerations

The study adhered to ethical guidelines and principles, receiving approval from the Scientific Research Ethics Committee at Zarqa University (Approval No. 54/2021/2022). Before participation, all subjects provided electronic informed consent, outlining the study’s purpose, voluntary nature, and the assurance of participant anonymity.

### Data Analysis

Statistical analyses were conducted using IBM SPSS software, version 27. Descriptive statistics were utilized for summarizing demographic data and attitude statement responses, including frequency (N), percentage (%), mean (M), and standard deviation (SD). Inferential statistical tests were employed to identify significant variations and associations within the study groups, including independent *t*-tests, one-way ANOVA, Pearson’s correlation (*r*), and logistic regression. Odds ratios (OR) and 95% confidence intervals (CI) were calculated for each predictor variable. The significance level was set at *p* < 0.05 for all statistical tests. Cronbach’s alpha was calculated to assess Likert-scale items’ internal consistency and reliability (0.902).

## Results

### Demographic Characteristics

A total of 702 pharmacy students, representing diverse demographic backgrounds, participated in the study. Most students were from Jordan (N = 255, 36.3%), followed by Libya (N = 136, 19.4%), Egypt (N = 126, 17.9%), Lebanon (N = 112, 16.0%), Palestine (N = 67, 9.5%), and Saudi Arabia (N = 6, 0.9%). The average age was 21.9 years (SD = 2.9), with 72.8% female and 27.2% male. Most participants were single (88.9%), and the distribution of monthly household income included 28.9% in the lower class, 36.6% in the middle class, and 34.5% in the upper class. The majority (87.9%) held the local nationality of their study country, while only 12.1% were international students.

### Education and Employment

Regarding education, the majority enrolled in governmental universities (55.6%), were pursuing a bachelor of pharmacy (53.3%), and were distributed across academic years. Cumulative GPA achievement reflected a diverse range, with 22.2% scoring excellent, 47.6% very good, 26.5% good, and 3.7% fair. Regarding employment status, 64.2% were not working, 17.8% were employed, and 17.9% were interns or trainees. Among those employed, the majority worked or trained in community pharmacies (76.1%), while 23.9% worked in other settings. Table 1 shows participants’ education-related information.

### Technology Literacy and AI Familiarity

Participants’ responses regarding tech-savviness (knowledgeable or skilled in utilizing contemporary technology, particularly computers) revealed that 38.0% were neutral about having this skill, followed by agreement (39.7%) and disagreement (22.2%). Also, familiarity with AI varied, with 34.6% being neutral, 40.1% disagreeing, and 25.4% agreeing.

### Perceived Barriers Regarding AI

**Figure 1:**
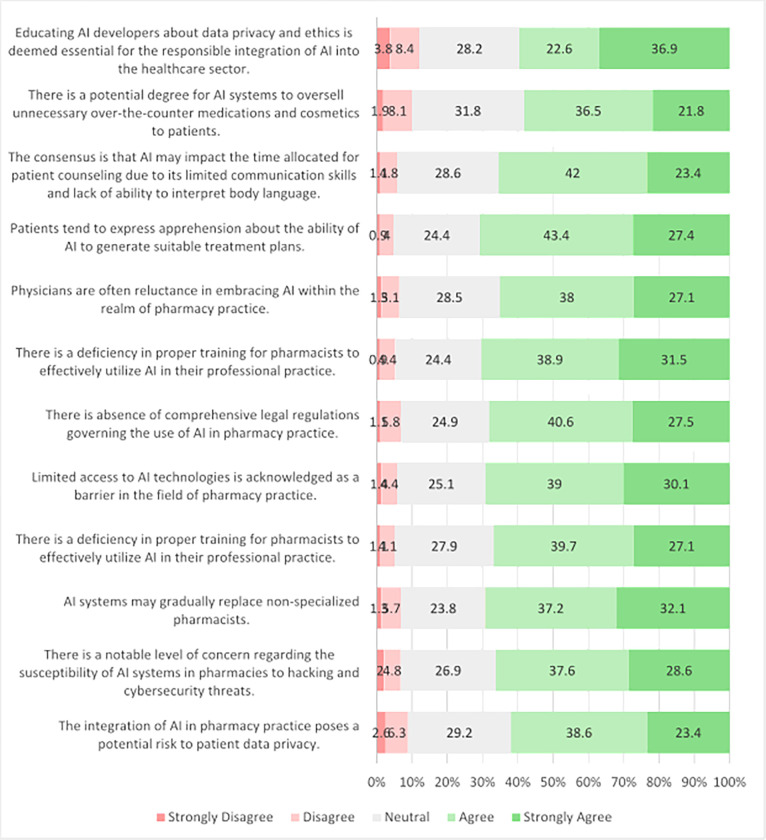
Student’s perceptions about AI-related issues in pharmacy practice (N=702).

Figure 1 shows participants’ perspectives on barriers and ethical issues related to AI integration in future pharmacy practice. Concerns about patient data privacy (38.6% agree, 23.4% strongly agree) and susceptibility to hacking (37.6% agree, 28.6% strongly agree) were notable. Participants were also concerned about AI replacing non-specialized pharmacists (37.2% agree, 32.1% strongly agree) and the cost limitations hindering its use (39.7% agree, 27.1% strongly agree). Access barriers (39.0% agree, 30.1% strongly agree) and the absence of comprehensive legal regulation (40.6% agree, 27.5% strongly agree) were also significant concerns. Participants emphasized the need for proper training (38.9% agree, 31.5% strongly agree) and expressed concerns about physicians’ reluctance (38.0% agree, 27.1% strongly agree) and patient apprehension (43.4% agree, 27.4% strongly agree). Concerns also included potential impacts on patient counseling time (42.0% agree, 23.4% strongly agree) and biased AI systems overselling medications (36.5% agree, 21.8% strongly agree). Educating AI developers about data privacy and ethics was deemed essential (22.6% agree, 36.9% strongly agree).

### Factors Influencing Attitudes

The total attitude score, calculated based on participant responses, had a mean of 3.9/5 (SD = 0.6), indicating a generally negative attitude among participants. Of 702 participants, 52.1% were higher than the mean score and exhibited a negative attitude, while 47.9% demonstrated a positive attitude toward AI integration in pharmacy practice. Tables 3 and 4 provide a detailed analysis of demographic parameters impacting the attitude score. Factors such as country of residence, age, university, academic year, cumulative GPA achievement, work status, technology literacy, and basic AI understanding significantly influenced students’ attitudes. Participants from private universities, those progressing in higher academic years, with a higher GPA, actively participate in the working environment, and individuals with higher tech-savviness and AI understanding reported a more negative attitude towards AI. Age exhibited a slight positive correlation with attitude, indicating a minor concern increase as age increased. Also, there is a positive correlation between the attitude score and tech-savviness (*r* = 0.174, *p* < 0.001) and basic AI understanding (*r* = 0.155, *p* < 0.001).

Table 5 highlights logistic regression analyses that investigated the impact of these various factors on the attitude score related to AI integration in the pharmaceutical field. Participants from the lower and middle classes had significantly higher odds of having a more negative attitude toward AI integration compared to those from the upper class. Second- and fourth-year students showed significantly higher odds of negative attitudes towards AI integration than rst-year students. Second-year students had the highest odds ratio (OR = 21.3, 95% CI: 1.14–396.85). Participants who disagreed with being tech-savvy had significantly lower odds of having a negative attitude towards AI integration than those who strongly agreed (OR = 0.2, 95% CI: 0.057–0.731).

## Discussion

Understanding the ethical implications of AI adoption in future pharmacy education and practice is evolving to inform discussions and policy about the persistent “digital native” concept surrounding technological proficiency and generational differences, particularly in the context of ethical considerations related to AI integration [14]. This study’s results shed light on pharmacy students’ perspectives regarding AI integration into their education and practice, mainly focusing on ethical considerations.

Several studies have explored the ethical integration of AI, particularly ChatGPT, in pharmacy education. They emphasized the importance of ethical education in curricula, the reliance on AI support among students, and the need for ethical guidelines. The development and validation of the KAP-C tool provide crucial insights into pharmacy students’ perspectives on AI [15]. However, there’s a noted paradox between AI benefits and concerns over privacy and human rights, emphasizing ongoing refinement for ethical AI integration. Findings underscore the need for ethical guidance and pedagogical strategies to ensure responsible AI use in pharmacy education [16]. Moreover, there’s a call for integrating ethical education into pharmacy curricula and establishing robust ethical guidelines to govern AI integration, highlighting the importance of ongoing discourse and refinement to align the AI in Education (AIED) systems with societal values [17]. To navigate ethical complexities, these systems propose frameworks highlighting seven core principles, including governance, transparency, sustainability, privacy, security, inclusiveness, and human-centered design.

Participants expressed various concerns and barriers related to AI integration in pharmacy. Notably, concerns regarding patient data privacy, susceptibility to hacking, potential replacement by AI systems, and cost limitations hindering accessibility were prominent. These concerns are consistent with previous research highlighting the importance of data privacy, cybersecurity, and equitable access to AI technologies in healthcare settings [18, 19]. Additionally, participants emphasized the need for comprehensive legal regulations governing the use of AI in pharmacy practice, proper training to utilize AI effectively, and addressing physicians’ reluctance and patient apprehension toward AI technologies. These findings underscore the importance of regulatory frameworks, education, and stakeholder engagement in fostering responsible AI integration in healthcare [20].

Research in various countries, including the UAE, Pakistan, Jordan, and Palestine, indicates positive attitudes of pharmacy and medical students towards AI in healthcare and education [21–25]. However, concerns remain about over-reliance, ethical implications, lack of awareness, and training persist. While students acknowledge the potential benefits of AI, they emphasize the importance of addressing ethical considerations, integrating AI education into curricula, and promoting responsible AI usage in academia and practice. This aligns with our findings on pharmacy students’ concerns about AI, suggesting a shared sentiment across healthcare disciplines regarding the potential benefits and challenges of integrating AI into education and practice.

Our study revealed a generally negative attitude among participants toward AI integration in pharmacy practice. Factors such as country of residence, monthly household income, university type, academic year, cumulative GPA achievement, work status, technology literacy, and basic AI understanding significantly influenced participants’ attitudes. These findings contradict a prior study suggesting that individuals with higher technology literacy may exhibit more positive attitudes toward AI [25]. This indicates a need for targeted interventions to address concerns and promote positive attitudes among pharmacy students, particularly those with higher academic achievement and technological proficiency.

These identified factors also reveal several noteworthy associations that provide insights into the dynamics of these concerns among pharmacy students. The discrepancy between countries might be influenced by diverse cultural, educational, or contextual factors in these regions. Interestingly, gender did not emerge as a significant predictor, suggesting that concerns about AI ethics are relatively uniform across male and female students. This did not align with our previous research, which indicated a different level of awareness and interest in AI-related topics among students [25]. The lack of a gender effect suggests a shared perception of the ethical implications of AI in pharmacy practice.

Monthly household net income was associated with students’ concerns, with those in the lower and middle-income brackets demonstrating a higher likelihood of expressing concerns. Economic factors can influence individuals’ perceptions of emerging technologies. Addressing these concerns is essential to ensure inclusivity and equal opportunities for students from diverse socioeconomic backgrounds. Academic factors, including university, academic year, and GPA, also shaped AI ethics concerns. These findings underline the need for targeted educational interventions, tailoring AI ethics discussions to students’ specific academic context and progression. Another notable aspect is the influence of technology literacy and AI understanding on concerns. Students who reported agreement with being tech-savvy expressed concerns, indicating a potential relationship between technological familiarity and reduced ethical apprehensions.

In considering the integration of AI into pharmacy education and practice, iťs crucial to identify the competencies that pharmacy graduates need to navigate and excel in an AI-driven world [26]. Pharmacy graduates must possess a blend of traditional pharmaceutical knowledge alongside technical competencies in data analytics and visualization, computational thinking, and ethical decision-making within AI applications in healthcare [27, 28]. Additionally, pharmacy graduates should be capable of collaborating effectively with multidisciplinary teams and be skilled communicators capable of effectively conveying complex AI-driven insights to patients and caregivers clearly and understandably [29, 30].

To ensure that these competencies are adequately addressed in pharmacy curricula, it is essential to integrate AI and digital health education throughout the pharmacy curriculum rather than treating them as standalone modules [31, 32]. Precisely, the integration can align with the Patient Care Process (PCPP) Framework, a foundational model that outlines the steps involved in providing pharmaceutical care [33]. AI can be incorporated into each stage of the PPCP Framework, from patient assessment and medication selection to monitoring and follow-up, to optimize clinical decision-making and improve patient outcomes.

Faculty and staff can leverage AI in pharmacy education to streamline administrative tasks, facilitate personalized learning experiences, and enhance teaching effectiveness [34]. Despite the potential benefits of AI in education, faculty and educational institutions must prioritize ethical AI practices and ongoing monitoring of AI systems’ impact on student learning outcomes [35]. Additionally, students should be educated about the ethical implications of AI use and encouraged to evaluate AI-driven recommendations in the context of patient care critically.

### Implications for Pharmacy Education and Practice:

There is a need to incorporate education on AI, digital health and biomedical ethics into pharmacy curricula, preparing future pharmacists with the skills and knowledge to navigate the ethical complexities of AI integration. Augmented reality, which combines virtual reality with AI, is pivotal in hybrid education and enhanced learning experiences, offering automation for teachers, personalized learning, and adaptive assessments [4]. This synergy creates personalized and immersive learning experiences, addressing individual students’ needs. Moreover, collaborative efforts involving stakeholders from various disciplines are essential to develop robust regulatory frameworks and promote responsible AI utilization in healthcare. Finally, regulatory bodies and policymakers should work to develop comprehensive legal frameworks and guidelines for the use of AI in pharmacy practice, addressing issues such as data privacy, accountability, and equity.

### Limitations of the Study and Future Research

The sample size may not represent the entire pharmacy student’s community, limiting the generalizability of the findings. Additionally, the survey relied on self-reported concerns, which could be subject to response bias. Future studies could bene t from more diverse and larger samples. Future research could conduct in-depth interviews to gain a more comprehensive understanding of the ethical concerns and perspectives of pharmacy professionals and stakeholders. Longitudinal studies could track changes in attitudes and concerns over time as AI becomes more integrated into pharmacy practice. Comparative studies across different countries could highlight variations in concerns and the readiness of varying healthcare systems for AI integration. They are investigating the effectiveness of specific interventions, such as education programs, in mitigating ethical concerns and improving the responsible implementation of AI in pharmacy practice.

Finally, this study provides valuable insights into pharmacy students’ ethical considerations and attitudes regarding AI integration in pharmacy practice. By addressing the identified concerns and promoting ethical AI practices, pharmacy education and practice can harness AI’s potential to enhance patient care, improve outcomes, and advance the profession.

## Conclusion

This study has revealed the multifaceted landscape of AI integration in pharmacy education and practice, underscoring significant concerns and potential benefits. The findings emphasize the pressing need for ethical guidelines and regulatory frameworks that protect patient data privacy, ensure cybersecurity, and promote equitable access to AI systems. As the pharmacy profession navigates the transformative power of AI, future research endeavors should focus on innovative solutions, educational strategies, and collaborative models that maximize the advantages of AI while safeguarding patient welfare and the ethical principles inherent to biomedical practice.

## Figures and Tables

**Table 1:** Education and employment of pharmacy students (N = 702).

Variables	Categories	N (%)
High school	Public school	452 (64.4)
	Private school	250 (35.6)
University	Governmental	390 (55.6)
	Private	312 (44.4)
Academic major	BPharm	374 (53.3)
	PharmD	308 (43.9)
	MSc	20 (2.8)
Academic year	First	28 (4.0)
	Second	59 (8.4)
	Third	186 (26.5)
	Fourth	256 (36.5)
	Fifth	134 (19.1)
	Sixth	19 (2.7)
	Master’s degree level	20 (2.8)
Cumulative GPA achievement	Excellent	156 (22.2)
	Very Good	334 (47.6)
	Good	186 (26.5)
	Fair	26 (3.7)
Work status	Not working	451 (64.2)
	Employment	125 (17.8)
	Internship or trainee	126 (17.9)
Workplace*	Community pharmacy	191 (76.1)
	Others	60 (23.9)

* This question allowed responses from those with active working status.

**Table 3:** Parameters affecting the mean total AI-attitudes score.

Variable	Categories	Mean ± SD	*p*-value*
Country of residence	Jordan	3.9 ± 0.6	**< 0.001**
	Egypt	3.9 ± 0.6	
	Lebanon	3.6 ± 0.7	
	Libya	3.8 ± 0.7	
	Palestine	3.8 ± 0.5	
	Saudi Arabia	4.1 ± 0.5	
Gender	Males	3.8 ± 0.7	0.169
	Females	3.9 ± 0.6	
Student Nationality	Local	3.8 ± 0.6	0.073
	International	4.0 ± 0.6	
Material status	Single	3.9 ± 0.6	0.996
	Married	3.9 ± 0.6	
Monthly household income	Lower class	3.9 ± 0.6	0.791
	Middle class	3.8 ± 0.7	
	Upper class	3.9 ± 0.6	
High school	Public	3.9 ± 0.6	0.156
	Private	3.8 ± 0.7	
University	Governmental	3.8 ± 0.7	**0.004**
	Private	3.9 ± 0.6	
Academic major	Bachelor’s	3.8 ± 0.6	0.374
	PharmD	3.9 ± 0.7	
Academic year	First and Second (Basics/Foundation)	3.6 ± 0.7	**< 0.001**
	Third (Intermediate)	3.8 ± 0.6	
	Fourth (Intermediate)	4.0 ± 0.6	
	Fifth (Intermediate)	3.9 ± 0.6	
	Sixth and Master’s level (Advanced/Specialized)	3.9 ± 0.6	
Cumulative GPA achievement	Excellent	4.0 ± 0.6	**0.002**
	Very good	3.9 ± 0.6	
	Good	3.8 ± 0.6	
	Fair	3.5 ± 0.9	
Work status	Not working	3.8 ± 0.7	**0.021**
	Employment	4.0 ± 0.6	
	Internship	3.8 ± 0.6	
Workplace	Community Pharmacy	3.9 ± 0.6	0.668
	Others	3.9 ± 0.6	
Tech-savviness	Strongly disagree	3.7 ± 0.7	**< 0.001**
	Disagree	3.7 ± 0.7	
	Neutral	3.8 ± 0.6	
	Agree	3.9 ± 0.6	
	Strongly agree	4.0 ± 0.6	
Basic AI understanding	Strongly disagree	3.7 ± 0.7	**0.002**
	Disagree	3.8 ± 0.6	
	Neutral	3.9 ± 0.6	
	Agree	4.0 ± 0.6	
	Strongly agree	4.0 ± 0.6	

* A *p*-value < 0.05 indicates statistical significance, calculated by an independent *t*-test or ANOVA when appropriate.

**Table 4:** Correlation analysis of independent variables with the total attitudes score.

Variable	M ± SD	Pearson Correlation Coefficient (*r*)	*p*-value*
Age	21.9 ± 2.9	0.089	**0.018**
Tech-savviness	3.3 ± 1.1	0.174	**< 0.001**
Basic AI understanding	2.8 ± 1.1	0.155	**< 0.001**
Total attitude score	3.9 ± 0.6	1	-

* A *p*-value < 0.05 indicates statistical significance, calculated by Pearson’s *r*.

**Table 5:** Factors associated with students’ attitudes towards AI.

Categories	Sub-Categories	OR	95% CI. for OR		*p*-value*
			Lower	Upper	
Country of residence	Jordan	0.943	0.295	3.009	0.920
	Libya	1.307	0.366	4.660	0.680
	Lebanon	0.523	0.137	1.990	0.342
	Egypt	0.830	0.218	3.156	0.784
	Palestine (REF)	-	-	-	0
Gender	Male	0.981	0.452	2.133	0.962
	Female (REF)	-	-	-	0
Student Nationality	Local	2.124	0.695	6.497	0.187
	International (REF)	-	-	-	0
Age		1.106	0.971	1.260	0.130
Marital status	Single	1.592	0.595	4.257	0.354
	Married (REF)	-	-	-	0
Monthly household income	Lower Class	2.840	1.091	7.390	**0.032**
	Middle Class	2.389	1.087	5.248	**0.030**
	Upper Class (REF)	-	-	-	0
High school	Public	1.024	0.466	2.253	0.952
	Private (REF)	-	-	-	0
University	Governmental	0.564	0.266	1.197	0.136
	Private (REF)	-	-	-	0
Academic major	BPharm	0.136	0.007	2.655	0.188
	PharmD	0.342	0.020	5.737	0.456
	MSc (REF)	-	-	-	0
Academic year	1st	0.000	0.000	-	1.000
	2nd	21.272	1.140	396.849	**0.041**
	3rd	7.340	0.538	100.213	0.135
	4th	16.402	1.257	214.007	**0.033**
	5th	7.306	0.509	104.833	0.143
	6th (REF)	-	-	-	0
Cumulative GPA achievement	Excellent	0.152	0.009	2.603	0.194
	Very good	0.130	0.008	2.154	0.154
	Good	0.060	0.003	1.022	0.052
	Fair (REF)	-	-	-	0
Work status	Not working	1.487	0.682	3.242	0.319
	Other (REF)	-	-	-	0
Workplace	Community Pharmacy	0.915	0.410	2.041	0.828
	Other (REF)	-	-	-	0
Tech-savviness	Strongly Disagree	0.720	0.063	8.179	0.791
	Disagree	0.205	0.057	0.731	**0.015**
	Neutral	0.385	0.148	0.999	0.050
	Agree	0.398	0.151	1.051	0.063
	Strongly Agree (REF)	-	-	-	0
Basic AI understanding	Strongly Disagree	1.257	0.254	6.211	0.779
	Disagree	1.253	0.311	5.049	0.751
	Neutral	1.430	0.396	5.160	0.585
	Agree	0.975	0.280	3.394	0.968
	Strongly Agree (REF)	-	-	-	0

* A *p*-value < 0.05 indicates statistical significance.

## Data Availability

The dataset supporting the conclusions of this article is included within the article and its additional file.

## Abbreviations

AI: Artificial intelligence
